# Fracture Behavior and Energy Analysis of 3D Concrete Mesostructure under Uniaxial Compression

**DOI:** 10.3390/ma12121929

**Published:** 2019-06-14

**Authors:** Yiqun Huang, Shaowei Hu, Zi Gu, Yueyang Sun

**Affiliations:** 1 College of Mechanics and Materials, Hohai University, Nanjing 210098, China; yiqunhuang@hhu.edu.cn; 2 School of Civil Engineering, Chongqing University, Chongqing 400044, China; albert_gu@foxmail.com; 3 College of Water Conservancy and Hydropower Engineering, Hohai University, Nanjing 210098, China; syyang0118@126.com

**Keywords:** meso, concrete, voronoi tessellation, cohesive element, compression, fracture

## Abstract

In order to investigate the fracture behavior of concrete mesostructure and reveal the inner failure mechanisms which are hard to obtain from experiments, we develop a 3D numerical model based on the Voronoi tessellation and cohesive elements. Specifically, the Voronoi tessellation is used to generate the aggregates, and the cohesive elements are applied to the interface transition zone (ITZ) and the potential fracture surfaces in the cement matrix. Meanwhile, the mechanical behavior of the fracture surfaces is described by a modified constitutive which considers the slips and friction between fracture surfaces. Through comparing with the experiments, the simulated results show that our model can accurately characterize the fracture pattern, fracture propagation path, and mechanical behaviors of concrete. In addition, we found that the friction on the loading surfaces has a significant effect on the fracture pattern and the strength of concrete. The specimens with low-friction loading surfaces are crushed into separate fragments whereas those with high-friction loading surfaces still remain relatively complete. Also, the strength of concrete decreases with the increase of the specimen height in the high friction-loading surfaces condition. Further, the energy analysis was applied to estimate the restraint impact of loading surfaces restraint on the compressive strength of concrete. It shows that the proportion of the friction work increases with the increase of the restraint degree of loading surfaces, which finally causes a higher compressive strength. Generally, based on the proposed model, we can characterize the complicated fracture behavior of concrete mesostructure, and estimate the inner fracture mode through extracting and analyzing the energies inside the cohesive elements.

## 1. Introduction 

Concrete is a composite material consisting of aggregates, cement matrix, interface transition zone (ITZ) and some other content such as pores. These mesoscopic components determine the macro mechanical behaviour of concrete. In order to research the effect of these mesoscopic components on the mechanical behaviors, researchers have presented many modelling and simulation methods.

In terms of the modelling, there are two typical methods to rebuild the concrete mesostructure. One is to generate the aggregates and pores in a random way such as randomly throwing circles/spheres [1,2,3,4] or polygon/polyhedrons [5,6,7,8], and the other is to directly restructure the internal components of concrete based on the tomography which are obtained from computed tomography (CT) scanning [9,10,11]. In terms of the simulation methods, previous works have proposed many effective numerical methods to research the rebuilt concrete including the traditional finite element method (FEM) with the continuing solid elements [12,13,14,15,16], the lattice model with the lattice elements [17,18,19,20,21], the homogeneous model with the homogenized elements for the heterogeneity of concrete [22,23,24,25,26], and the improved FEM method with cohesive elements [27,28,29,30,31,32,33,34,35]. Besides the aforementioned FEM methods, the discrete element method (DEM) with separated elements such as the particle flow method [36,37,38] and rigid-body-spring method (RBSM) [39,40,41] is also applied in simulation of concrete. 

In recent years, more researchers have adopted the model with cohesive elements to simulate the fracture process of concrete [31,32,33,34,35]. As a transitional element inserted into the edges of solid elements [42], the cohesive element can set its geometric thickness as any non-negative value including zero, and it can only transfer the normal stress and shear stress. Due to the aforementioned characteristics, the cohesive element is very suitable to characterize the mechanical behavior of the interfaces with extremely small thickness such as ITZ, and it is also applicable for the representation of the potential/existing fracture surfaces. In addition, previous works indicated that the model with cohesive elements is size independent in a reasonable range of mesh size [33,34,35], which means the extremely fine mesh is unnecessary. Hence, the cohesive element can save significant calculation time and space. However, existing research about the cohesive elements mainly focused on the tensile fracture behavior of concrete [30,31,32,33,34,35], and only a few studies were about the fracture behavior under compression [28]. In these studies, the friction effect is rarely considered. Some only considered the tensile and shear damage without the consideration of friction in Refs. [33,34,35], and the other only considered the friction as an enhancement of shear stress in Refs. [28,29,30]. However, the fracture behavior of concrete under compression is pretty complicated. It contains almost all fracture modes which may occur inside the concrete including the tensile-shear fracture, the compressive-shear fracture, the separation and close of cracks, and the friction and slips between fracture surfaces. For this reason, a more appropriate constitutive of cohesive elements should be established to characterize the complicated fracture behavior of concrete accurately.

The fracture behaviour of concrete under uniaxial compression has been studied by several methods, which includes the traditional FEM method such as the homogeneous model [22] and the lattice model [20,21], and the DEM method such as rigid-body-spring methods [40,41] and particle flow [37,38]. These researches have provided some valuable results and conclusions such as the effect of aggregates content and loading condition on the mechanical behavior of concrete. Based on these researches and the cohesive element model mentioned above, we can establish a more effective numerical model to investigate and reveal the inner failure mechanisms of concrete under various loading condition including compression.

In this paper, we present a 3D numerical model based on 3D Voronoi tessellation and zero thickness cohesive elements. Based on the modified constitutive that takes into account the effect of the norm-tangential coupling and the friction between cracks, the proposed model can characterize the complicated fracture behavior of concrete mesostructure. Moreover, this model can also be used to estimate the inner fracture mode through extracting energies inside the cohesive elements. The fracture process of concrete specimens under compression is simulated through the ABAQUS [42] with subroutine VUMAT that the modified constitutive is implemented in. Then, we investigate the effect of the loading surfaces friction and the specimen height on the fracture behavior of concrete under uniaxial compression. Finally, the restraint influence of loading surfaces is estimated by the energy analysis.

## 2. Numerical Model

### 2.1. Generation of Aggregates Based on 3D Voronoi Tessellation

**Figure 1:**
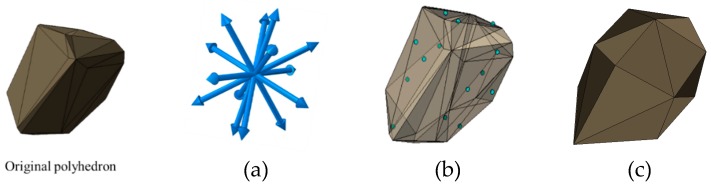
The simplified process of the polyhedron: (**a**) determine the pre-set vectors starting from the center of the polyhedron, (**b**) find the intersection points between the vectors and surfaces, (**c**) obtain the new surfaces of the polyhedron by determining the convex hull of intersection points.

Based on Voronoi polyhedron tessellation [43,44,45], a 3D aggregates generation method is presented in this section. The Voronoi tessellation can generate seamlessly connected cells according to the seeds that were placed randomly in advance within a given region. Normally, one seed can generate one corresponding polyhedron. Thus, we can generate aggregates by reasonably throwing seeds and reducing the size of generated polyhedrons. In this paper, we obtain seeds by randomly generating the spheres without overlapping. Also, it is noted that the generated polyhedrons normally have too many vertices and surfaces, which could cause great difficulties in modeling. For this reason, simplifying the vertices’ number of polyhedrons is necessary. The method to reduce the vertices number of polyhedrons is established on finding the intersection points between the pre-set vectors and the surfaces of polyhedrons, and the new surfaces of polyhedrons can be obtained by determining the convex hull of the reduced vertices, as shown in Figure 1. The pre-set vectors starting from the center of polyhedron are generated by rotating the basic vectors randomly. In this paper, the basic vectors are {[1,0,0], [0,1,0], [1,1,0], [−1,1,0], [0,0,1], [1,1,1], [−1,1,1], [−1−1,1], [1,−1,1], [0,1,1], [1,0,1]}, which means 22 intersection points can be obtained by these 11 vectors.

**Figure 2:**
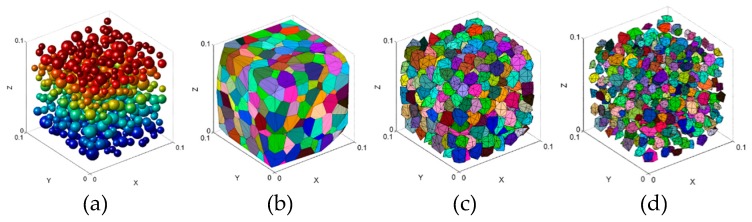
The generating process of the aggregates in concrete: (**a**) throw the spheres randomly in a specified region and extract the spheres centers as the seeds, (**b**) generate the Voronoi polyhedrons based on extracted seeds, (**c**) simplify the generated polyhedrons, (**d**) obtain the aggregates by zooming simplified polyhedrons.

On the basis of the simplified polyhedrons, the whole process (Figure 2) to generate the aggregates can be written as follows:

Step1: randomly throw the spheres in a specified region (the spheres should have no overlap and satisfy the size distribution of aggregates), and then take the centers of the spheres as the seeds to generate Voronoi polyhedrons.

Step2: generate the Voronoi polyhedrons based on the generated seeds.

Step3: simplify the polyhedrons by reducing the vertices and determine the convex hull of the reduced vertices.

Step4: zoom the simplified polyhedrons through the scaling ratio of the sphere volume to the polyhedron one which shares the same seed. The scaling ratio is normally less than 1 (in rare cases the ratio may greater than 1, and the ratio would be set to 1). The ratio can be calculated by:

(1) $$ratio=\{\begin{array}{lll} \sqrt[{3}]{\frac{V_{s}}{V_{d}}}\cdot c & \;\mathrm{if}\; & \sqrt[{3}]{\frac{V_{s}}{V_{d}}}\leq 1\\ 1 & \;\mathrm{if}\; & \sqrt[{3}]{\frac{V_{s}}{V_{d}}}>1 \end{array},$$

where $V_{s}$ is the volume of the sphere, $V_{d}$ is the volume of the simplified polyhedron, and c is an empirical correction factor whose range is 1 to 1.1.

### 2.2. Application of Cohesive Elements

**Figure 3:**
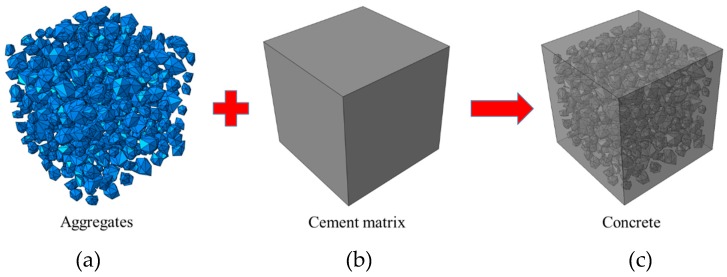
Generation of the two-phase mesostructure of concrete: (**a**) aggregates, (**b**) cement matrix, (**c**) concrete merged by aggregates and cement matrix.

After the generation of aggregates in concrete, a two-phase mesostructure of concrete can be generated easily by merging the cement matrix and aggregates as shown in Figure 3. Moreover, ITZ is an important component of the concrete mesostructure, it should be also considered in the model. There are two problems for traditional continuing solid elements. First, the solid element is hard to represent the ITZ whose thickness is far less than the size of aggregates. Secondly, the solid element is hard to characterize the behavior of the fracture surfaces in concrete. In order to solve these problems, the cohesive elements are inserted into the interfaces of the solid elements when we build the model of concrete mesostructure. The unique advantages of the cohesive element are that it only transfers the normal stress and shear stress and its geometry thickness is independent of its calculation thickness. These characteristics make the cohesive element can be used for the characterization of ITZ and potential fracture surfaces in concrete. In our model, the geometry thickness of the cohesive elements is set to 0 and the calculation one is set to 1, which means the behavior of ITZ and potential fracture surfaces in concrete are characterized by the stress–displacement relationship.

**Figure 4:**
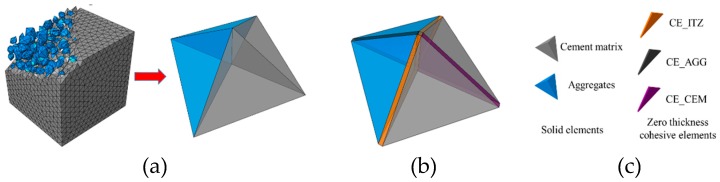
Insertion of the cohesive elements into the interfaces of the solid elements: (**a**) initial mesh of the two-phase structure, (**b**) the mesh with zero thickness cohesive elements, (**c**) element types in the mesh.

Based on the method in Ref. [35], the zero thickness cohesive elements are inserted into the interfaces of the solid elements in the initial mesh of the two-phase structure, and the process of inserting the cohesive elements is shown in Figure 4. Three types of zero cohesive elements exist in the model: (1) CE_ITZ, the cohesive element inserted into the interface of the aggregate and cement matrix; (2) CE_AGG, the cohesive element inserted into the interface inside the aggregate; (3) CE_CEM, the cohesive element inserted into the interface inside the cement matrix. Specially, because the aggregate strength is much higher than the mortar and ITZ, we assume that the aggregate will not be damaged. Thus, CE_AGG is not considered in this paper. 

### 2.3. Modified Constitutive Applied in Cohesive Elements

The none-linear mechanical behavior is defined in the cohesive elements according to the previous work [27,28,29,30,31,32,33,34,35]. Thus, a reasonable and effective constitutive relationship to the cohesive elements is the basis of obtaining accurate numerical results. For the tensile condition, the constitutive can be obtained easily by determining the relation between the normal stress and normal displacement without the consideration of the shear stresses and friction stress. However, the shear stresses and friction stress are important in compression condition. For this reason, a reasonable constitutive which can characterize the normal stress, shear stresses and friction stress is needed for the simulation of the concrete under compression condition. 

A modified constitutive with the consideration of the friction stress is established based on the bilinear damage model. The constitutive is given by the relation of stresses and displacements. In the case of 3D model, one normal stress ($t_{n}$) and two orthogonal shear stresses ($t_{s}$,$t_{t}$) should be determined in the constitutive. 

In the stresses and displacements relation, two phases exist: (1) elastic phase, (2) damage phase. In elastic phase, the relation between the stresses and displacements can be expressed as:

(2) $$t_{n}=\bar{k_{n}}\cdot \delta_{n}\;,\;t_{s}=\bar{k_{s}}\cdot \delta_{s}\;,\;t_{t}=\bar{k_{s}}\cdot \delta_{t}\;,$$

where $\bar{k_{n}}$ is the initial stiffness of the normal direction, $\bar{k_{s}}$ is the initial stiffness of the tangential direction, $\delta_{n}$ is the normal displacement, $\delta_{s}$ is the tangential displacement in first direction, and $\delta_{t}$ is the tangential displacement in second direction.

The element initiates the damage phase (the potential fracture surface begins to develop) once:

(3) $${\left(\frac{\langle t_{n}\rangle }{t_{n0}}\right)}^{2}+{\left(\frac{t_{s}}{t_{s0}}\right)}^{2}+{\left(\frac{t_{t}}{t_{s0}}\right)}^{2}\geq 1\;,$$

where $t_{n0}$ is the tensile strength, $t_{s0}$ is the shear strength, and 〈x〉 is the Macaulay bracket, which can be expressed as:

(4) $$\langle x\rangle =\left\{ \begin{array}{l} x\;\mathrm{if}\;x>0\\ 0\;\mathrm{if}\;x\leq 0 \end{array}\right.$$

**Figure 5:**
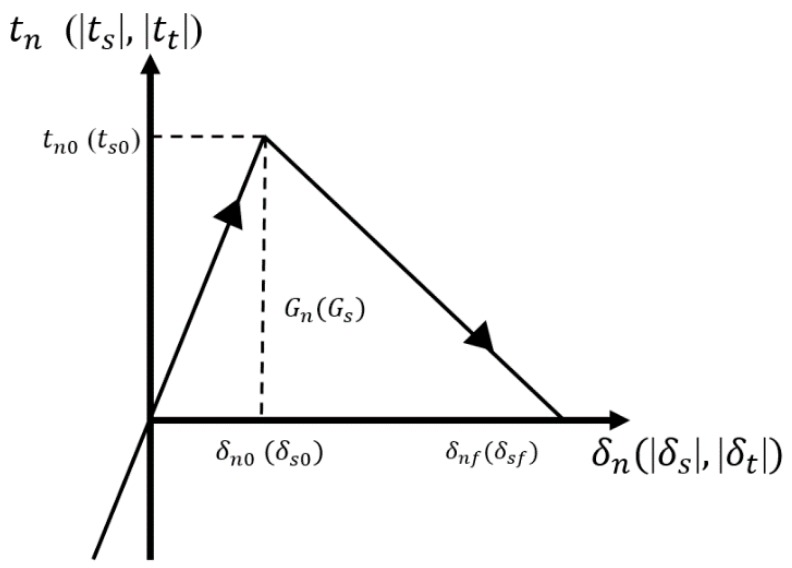
Stress–displacement relationship in a single direction.

The stress-displacement relationship in a single direction is shown in Figure 5. The area under the curve represents the normal fracture energy $G_{n}$ (tangential fracture energy $G_{s}$). The fracture energies can be calculated by:

(5) $$G_{n}=\frac{\delta_{nf}t_{n0}}{2},G_{s}=\frac{\delta_{sf}t_{s0}}{2}\;,$$

where $\delta_{nf}$ is the failure displacement in normal direction, and $\delta_{sf}$ is the failure displacement in tangential direction.

The normal (tangential) initial damage displacement $\delta_{n0}$ ($\delta_{s0}$) in Figure 5 can be expressed by:

(6) $$\delta_{n0}=\frac{t_{n0}}{{\bar{k}}_{n}}\;,\;\delta_{s0}=\frac{t_{s0}}{{\bar{k}}_{s}}$$

**Figure 6:**
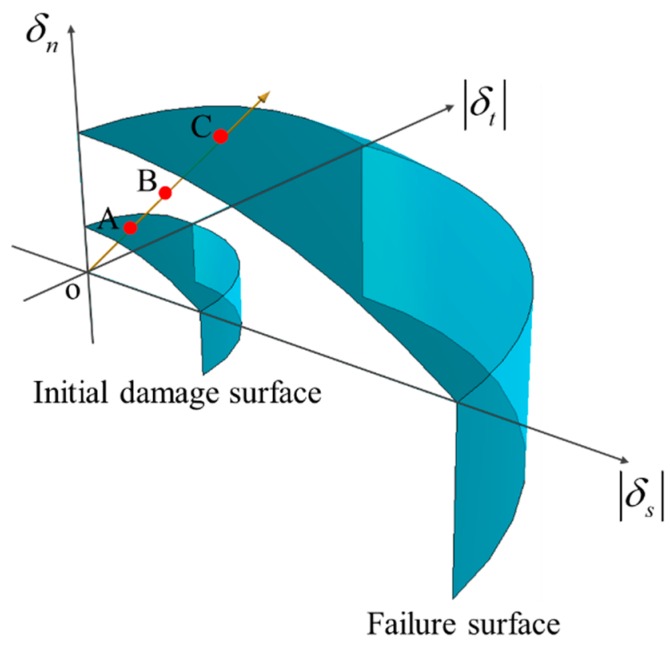
Initial damage and failure surfaces, and the relative displacement points: point B as the historic maximum displacement point, point A as the intersection point of vector OB and the initial damage surface, point C as the intersection point of vector OB and the failure surface.

The initial damage and failure surfaces controlled by normal and tangential displacements are established (Figure 6), which can be calculated by:

(7) $$\begin{array}{l} {\left(\frac{\langle \delta_{n}\rangle }{\delta_{n0}}\right)}^{2}+{\left(\frac{\delta_{s}}{\delta_{s0}}\right)}^{2}+{\left(\frac{\delta_{t}}{\delta_{s0}}\right)}^{2}=1\;(\mathrm{initial}\;\mathrm{damage}\;\mathrm{surface})\;,\\ {\left(\frac{\langle \delta_{n}\rangle }{\delta_{nf}}\right)}^{2}+{\left(\frac{\delta_{s}}{\delta_{sf}}\right)}^{2}+{\left(\frac{\delta_{t}}{\delta_{sf}}\right)}^{2}=1\;(\mathrm{failure}\;\mathrm{surface}) \end{array}$$

As shown in Figure 6, the point B ($\delta_{n,max}$, $\delta_{s,max}$, $\delta_{t,max}$) is the historic maximum displacement point, and $\sqrt{\delta_{n,max}{}^{2}+\delta_{s,max}{}^{2}+\delta_{t,max}{}^{2}}$ is the historic maximum displacement during the loading process. $\delta_{n,max}$, $\delta_{s,max}$, $\delta_{t,max}$ are the normal and two orthogonal tangential components of the historic maximum displacement respectively. The point A ($\delta_{n,0}$, $\delta_{s,0}$, $\delta_{t,0}$) is the relative initial damage displacement point, which can be obtained by finding the intersection point of vector OB and the initial damage surface. The point C ($\delta_{n,f}$, $\delta_{s,f}$, $\delta_{t,f}$) is the relative failure displacement point, and it is the intersection point of vector OB and the failure surface. The coordinate value of the point A can be calculated through the geometry relation in Figure 6:

(8) $$\begin{array}{l} \delta_{n,0}=\frac{\langle \delta_{n,max}\rangle \delta_{n0}\delta_{s0}}{\sqrt{\delta_{n0}{}^{2}(\delta_{s,max}{}^{2}+\delta_{t,max}{}^{2})+{\langle \delta_{n,max}\rangle }^{2}\delta_{s0}{}^{2}}}\;,\;\\ \delta_{s,0}=\frac{\delta_{s,max}\delta_{n0}\delta_{s0}}{\sqrt{\delta_{n0}{}^{2}(\delta_{s,max}{}^{2}+\delta_{t,max}{}^{2})+{\langle \delta_{n,max}\rangle }^{2}\delta_{s0}{}^{2}}}\;,\;\\ \delta_{t,0}=\frac{\delta_{t,max}\delta_{n0}\delta_{s0}}{\sqrt{\delta_{n0}{}^{2}(\delta_{s,max}{}^{2}+\delta_{t,max}{}^{2})+{\langle \delta_{n,max}\rangle }^{2}\delta_{s0}{}^{2}}} \end{array}$$

The coordinate value of the point C can also be similarly calculated by:

(9) $$\begin{array}{l} \delta_{n,f}=\frac{\langle \delta_{n,max}\rangle \delta_{nf}\delta_{sf}}{\sqrt{\delta_{nf}{}^{2}(\delta_{s,max}{}^{2}+\delta_{t,max}{}^{2})+{\langle \delta_{n,max}\rangle }^{2}\delta_{sf}{}^{2}}}\;,\\ \delta_{s,f}=\frac{\delta_{s,max}\delta_{nf}\delta_{sf}}{\sqrt{\delta_{nf}{}^{2}(\delta_{s,max}{}^{2}+\delta_{t,max}{}^{2})+{\langle \delta_{n,max}\rangle }^{2}\delta_{sf}{}^{2}}}\;,\\ \delta_{t,f}=\frac{\delta_{t,max}\delta_{nf}\delta_{sf}}{\sqrt{\delta_{nf}{}^{2}(\delta_{s,max}{}^{2}+\delta_{t,max}{}^{2})+{\langle \delta_{n,max}\rangle }^{2}\delta_{sf}{}^{2}}} \end{array}$$

So the relative displacements can be expressed as:

(10) $$\begin{array}{c} \delta_{max}=\sqrt{\delta_{n,max}{}^{2}+\delta_{s,max}{}^{2}+\delta_{t,max}{}^{2}}\;,\\ \delta_{0}\;=\sqrt{\delta_{n,0}{}^{2}+\delta_{s,0}{}^{2}+\delta_{t,0}{}^{2}},\\ \delta_{f}=\sqrt{\delta_{n,f}{}^{2}+\delta_{s,f}{}^{2}+\delta_{t,f}{}^{2}}, \end{array}$$

where $\delta_{max}$ is the relative historic maximum displacement, $\delta_{0}$ is the relative initial damage displacement, and $\delta_{f}$ is the relative failure displacement. 

According to the bilinear damage model in Figure 5, the damage factor D can be rewritten as follows:

(11) $$D=\frac{(\delta_{max}-\delta_{0})\delta_{f}}{(\delta_{f}-\delta_{0})\delta_{max}}$$

During the damage phase in the loading process, the friction stresses would occur when the cohesive element (fracture surface) is under compressive conditions. In the damaged area, the friction stress $T_{f}$ can be calculated by:

(12) $$T_{f}=\{\begin{array}{lll} \tau_{max}\cdot \frac{\delta_{def}}{\left|\delta_{def}\right|} & \;\mathrm{if}\; & \tau_{max}\leq {\bar{k}}_{s}\cdot \left|\delta_{def}\right|\\ {\bar{k}}_{s}\cdot \delta_{def} & \;\mathrm{if}\; & \tau_{max}>{\bar{k}}_{s}\cdot \left|\delta_{def}\right|\; \end{array}，$$

where $\tau_{max}$ is the maximum static friction stress which controls the calculation of $T_{f}$. When $\tau_{max}$ cannot prevent the relative slip of crack, $T_{f}$ should be calculated by the first formula in Equation (12). When the relative slip of the crack is prevented, $T_{f}$ can be calculated by the second formula. According to the friction law, $\tau_{max}$ can be calculated by:

(13) $$\tau_{max}=f\cdot \;{\bar{k}}_{n}\cdot \langle -\delta_{n}\rangle \;,$$

where f is the internal friction coefficient.

The tangential deformation displacement $\delta_{def}$ in Equation (12) can be calculated through two orthogonal tangential displacements ($\delta_{s}$,$\delta_{t}$) and two orthogonal slip displacements ($\delta_{s,slip}$, $\delta_{t,slip}$):

(14) $$\delta_{def}=\sqrt{{\left(\delta_{s}-\delta_{s,slip}\right)}^{2}+(\delta_{t}-\delta_{t,slip}{)}^{2}}$$

After the calculation of the friction stress, the orthogonal slip displacements should be updated when the slip of the crack occurs:

(15) $$\begin{array}{l} \delta_{s,slip}^{new}=\delta_{s}-\frac{\tau_{max}}{{\bar{k}}_{s}}\frac{\delta_{s}-\delta_{s,slip}^{old}}{\sqrt{{\left(\delta_{s}-\delta_{s,slip}^{old}\right)}^{2}+{\left(\delta_{t}-\delta_{t,slip}^{old}\right)}^{2}}}\;(\tau_{\max}\leq {\bar{k}}_{s}\delta_{def})\;,\\ \delta_{t,slip}^{new}=\delta_{t}-\frac{\tau_{max}}{{\bar{k}}_{s}}\frac{\delta_{t}-\delta_{t,slip}^{old}}{\sqrt{{\left(\delta_{s}-\delta_{s,slip}^{old}\right)}^{2}+{\left(\delta_{t}-\delta_{t,slip}^{old}\right)}^{2}}}\;(\tau_{\max}\leq {\bar{k}}_{s}\delta_{def})\;, \end{array}$$

where $\delta_{s,slip}^{new}$, $\delta_{t,slip}^{new}$ is the updated slip displacements, and $\delta_{s,slip}^{old}$, $\delta_{t,slip}^{old}$ is the slip displacements before the update.

According to the direction of the tangential deformation, the frictional stress components ($T_{f,s}$, $T_{f,t}$) in two orthogonal direction can be rewritten as follows:

(16) $$T_{f,s}=T_{f}\frac{\delta_{s}-\delta_{s,slip}^{old}}{\sqrt{{\left(\delta_{s}-\delta_{s,slip}^{old}\right)}^{2}+{\left(\delta_{t}-\delta_{t,slip}^{old}\right)}^{2}}}\;,\;T_{f,t}=T_{f}\frac{\delta_{t}-\delta_{t,slip}^{old}}{\sqrt{{\left(\delta_{s}-\delta_{s,slip}^{old}\right)}^{2}+{\left(\delta_{t}-\delta_{t,slip}^{old}\right)}^{2}}}$$

Finally, the stresses in the damage phase can be calculated by:

(17) $$\begin{array}{l} t_{n}=\left\{ \begin{array}{ccc} (1-D)\bar{k_{n}}\delta_{n} & \;\mathrm{if}\; & \delta_{n}>0\;\\ \bar{k_{n}}\delta_{n} & \;\mathrm{if}\; & \delta_{n}\leq 0 \end{array}\right.\;,\\ \begin{array}{l} t_{s}=(1-D)\bar{k_{s}}\delta_{s}+D\cdot T_{f,s}\;,\\ t_{t}=(1-D)\bar{k_{s}}\delta_{t}+D\cdot T_{f,t} \end{array} \end{array}$$

### 2.4. Calculation of Three Different Types of Energy

To analyze the fracture characteristic of the concrete, we extract the energies inside the cohesive elements during the loading process. Because three different kinds of stress (normal stress, tangential stress, and friction stress) exist in the cohesive element, three corresponding energies should be extracted respectively. These three energies are: (1) the normal stress work $Ed_{n}$ caused by the normal stress, (2) the shear stress work $Ed_{s}$ caused by the pure shear stress, (3) the friction stress work $Ed_{f}$ caused by the friction stress. These energies can be calculated by:

(18) $$\begin{array}{l} Ed_{n}=\iint_{A}(\int_{0}^{\delta_{n}}t_{n}d\delta )dA\;,\\ Ed_{s}=\iint_{A}(2\int_{0}^{\delta_{s}}(t_{s}-D\cdot T_{f,s})d\delta )dA+\iint_{A}(2\int_{0}^{\delta_{t}}(t_{t}-D\cdot T_{f,t})d\delta )dA\;,\\ Ed_{f}=\iint_{A}(2\int_{0}^{\delta_{s}}D\cdot T_{f,s}d\delta )dA+\iint_{A}(2\int_{0}^{\delta_{t}}D\cdot T_{f,t}d\delta )dA\;, \end{array}$$

where A is the area of the cohesive element.

The energies of the cohesive elements in whole concrete structure can be calculated through:

(19) $$\begin{array}{l} E_{n}=\sum_{cohesive\;elements}Ed_{n}\;,\\ E_{s}=\sum_{cohesive\;elements}Ed_{s}\;,\\ E_{f}=\sum_{cohesive\;elements}Ed_{f}\;, \end{array}$$

where $E_{n}$ ($E_{s}$, $E_{f}$) is the normal (shear, friction) stress work of the cohesive elements in whole concrete structure.

## 3. Numerical Examples and Results

### 3.1. Input Data of the Finite Element Model

**Figure 7:**
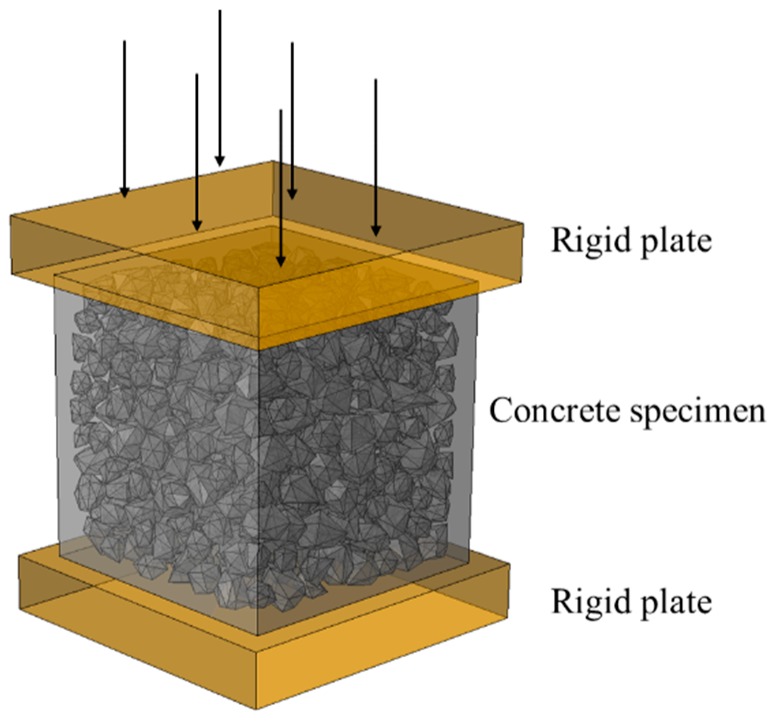
Loading scheme of the concrete specimen.

Square concrete specimens of 10 cm side are chosen for simulation. The density of the aggregates is assumed as 2800 kg/m^3^, and the gradation of aggregate size distribution based on Ref. [46] is listed in Table 1. To save the computing resources, only coarse aggregates larger than 2 mm are considered in this study. The loading scheme of the concrete specimen is shown in Figure 7. The specimen is placed between two rigid plates with the lower plate being constrained and the other plate being applied by a vertical displacement loading. The interaction between the concrete and the rigid plate includes normal contact and tangential friction. According to the friction coefficient of the interface between the concrete and the rigid plate, the loading condition can be divided into low-friction (LF) condition with the friction coefficient = 0.001 and high-friction (HF) condition with the friction coefficient = 0.2.

**Figure 8:**
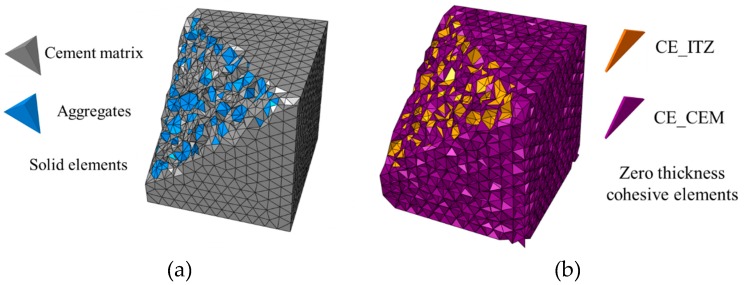
Mesh of the concrete specimen: (**a**) solid elements, (**b**) zero thickness cohesive elements.

Without the consideration of interfaces inside the aggregates, the mesh of the concrete specimen is shown in Figure 8. The previous works [33,34,35] have proved that the model with zero thickness cohesive elements is size-independent in a reasonable range. Thus, the model in this paper is meshed in a reasonable size (average element size = 5 mm) based on the repeat trial. One typical model has about 200,000 nodes, 60,000 solid tetrahedron elements and 150,000 zero thickness cohesive elements. It takes about 10 h to solve one model by parallel computation using 6 Intel I7 8700k CPUs @3.7Hz (School of Civil Engineering, Chongqing University, Chongqing, China).

According to the previous works about cohesive element model [28,29,30], repeated trial calculations, and the experimental results [46], the material parameters value applied in the solid elements are *E* = 70 GPa, *v* = 0.2, *ρ* = 2800 kg/m^3^ for aggregate; *E* = 25 GPa, *v* = 0.2, *ρ* = 2400 kg/m^3^ for cement matrix. The material parameters value applied in the cohesive elements of concrete are $\bar{k_{n}}$ = $\bar{k_{s}}$= 10^6^ GPa, *ρ* = 2400 kg/m^3^, $t_{n0}$ = 3.0 MPa, $t_{s0}$ = 10.5 MPa, $G_{n}$ = 40 N∙m, $G_{s}$ = 400 N∙m, *f* = 0.45 for cement matrix; $\bar{k_{n}}$ = $\bar{k_{s}}$ = 10^6^ GPa, *ρ* = 2400 kg/m^3^, $t_{n0}$ = 1.5 MPa, $t_{s0}$ = 5.25 MPa, $G_{n}$ = 20 N∙m, $G_{s}$ = 200 N∙m, *f* = 0.45 for ITZ. 

The simulations all end when the displacement *d* = 1 mm, corresponding to the macro strain *ε* = 0.01. The models are solved in ABAQUS/EXPLICIT solver, with the user subroutine VUMAT [42] in which the modified constitutive is implemented. According to the repeat trial calculation, the loading time is set to 1s which can ensure the quasi-static loading conditions. The cracks are represented by deleting the zero thickness cohesive elements whose damage coefficient is equal to 1. All specimens with random aggregates all show a similar result. For this reason, one typical specimen is chosen to be discussed in the next section. 

### 3.2. Effect of Loading Surface Friction Coefficient under Compression

#### 3.2.1. Low-Friction (LF) Condition

**Figure 9:**
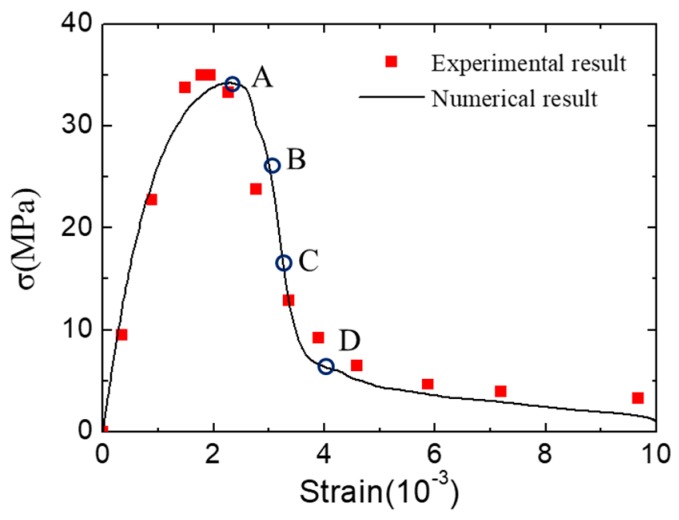
Average stress–strain curve of concrete under uniaxial compression in low-friction (LF) condition.

The numerical experiments of concrete under uniaxial compression in the LF condition are carried out. The average stress–strain curve of concrete is shown in Figure 9. The numerical result is similar to the experimental one [46] in terms of peak stress and the curve shape. To analyze the fracture patterns of concrete during the loading process in the LF condition, four typical states are chosen and marked in the stress–strain curve (Figure 9).

**Figure 10:**
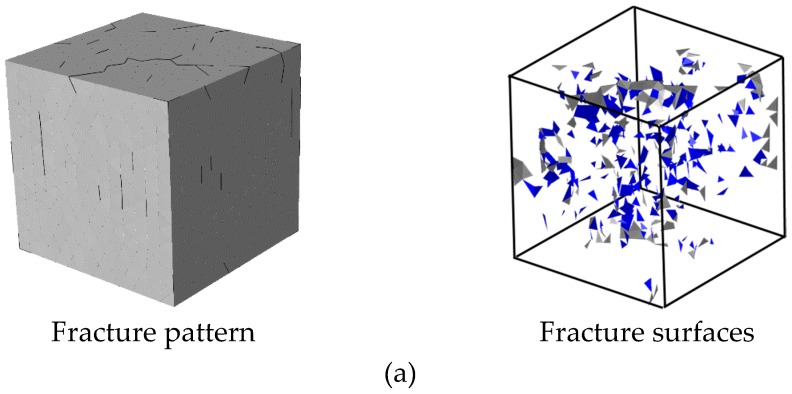

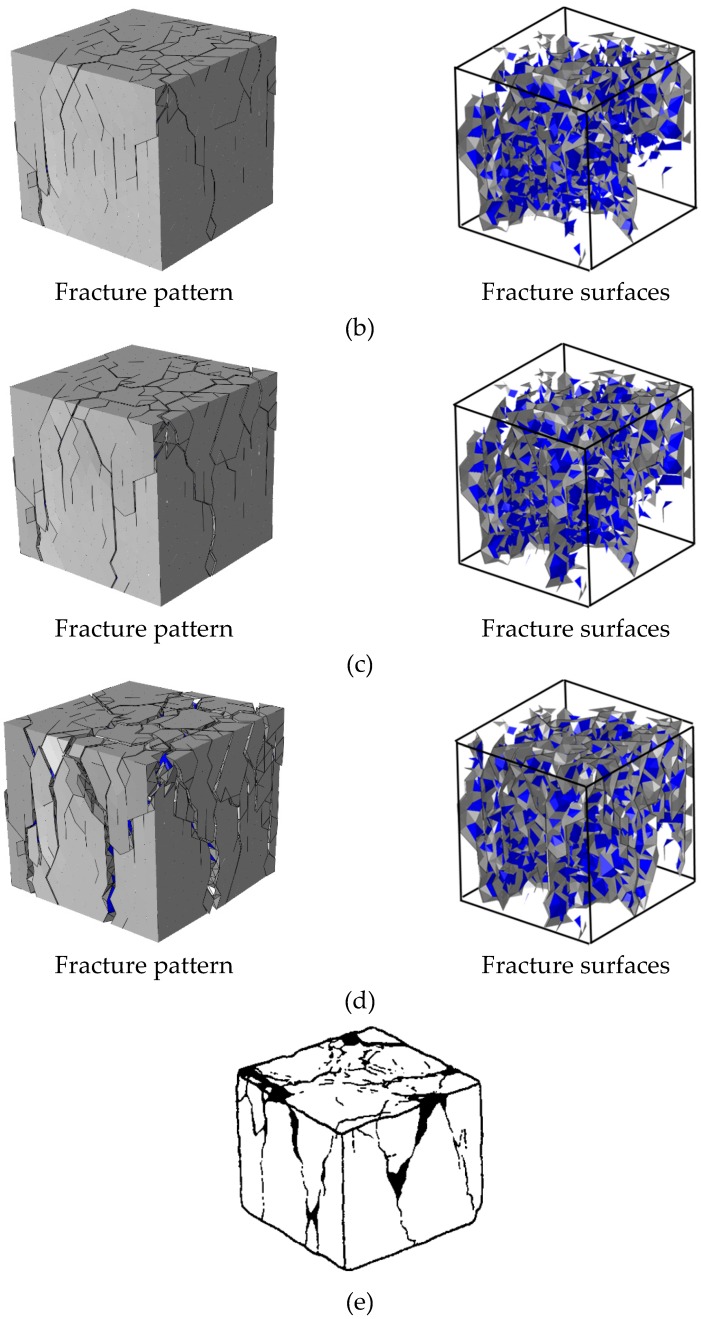
Fracture pattern (aggregates in blue and cement matrix in gray) and fracture surfaces (interface transition zone (ITZ) in blue and cement matrix interfaces in gray) in different states during the loading process in Figure 9: (**a**) state A, (**b**) state B, (**c**) state C, (**d**) state D and (**e**) the final fracture pattern in experiments [46] (License number: 4607510939744).

Figure 10 shows the fracture pattern (deformation is magnified 5 times) and fracture surfaces of concrete in four chosen states. At the initial state of failure, the cracks start developing by ripping through the loading surfaces and ITZ. With the increase of displacement loading, the cracks expand continually, and the opening degree of cracks increase either. It can be seen that the fracture surfaces always remain substantially parallel to the loading direction during the loading process. Finally, the specimen is penetrated by cracks and divided into several parts. The final fracture pattern of the numerical result shows an agreement to the experimental one.

**Figure 11:**
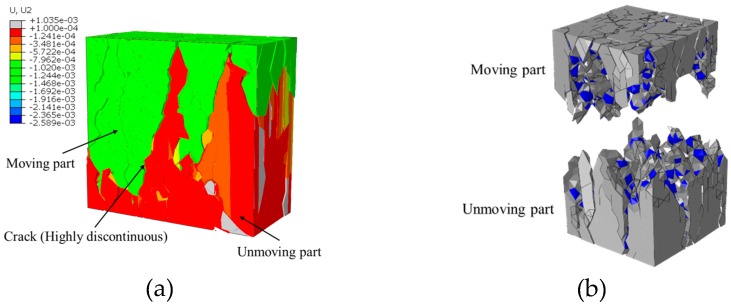
Two main parts of concrete divided through the distribution of the loading-direction displacement: (**a**) the loading-direction displacement nephogram of the concrete inner section, (**b**) the moving part and unmoving part.

More details of the final fracture pattern can be obtained from the loading-direction displacement nephogram (Figure 11a) of the concrete inner section at the final fracture status. The concrete can be divided into two parts according to the displacement value. One, namely the moving part, has a larger displacement value (the absolute value is greater than 1e^−3^) and is shown in light blue (Figure 11a), and the other, namely the unmoving part, has a small displacement value (the absolute value is smaller than 1.2e^−4^) and is shown in red. These two parts are separated by cracks with highly discontinuous. Thus, we can obtain the shape of these two parts by extracting the elements with the displacement value higher than 1e^−3^ and smaller than 1.2e^−4^ in the loading direction (Figure 11b).

**Figure 12:**
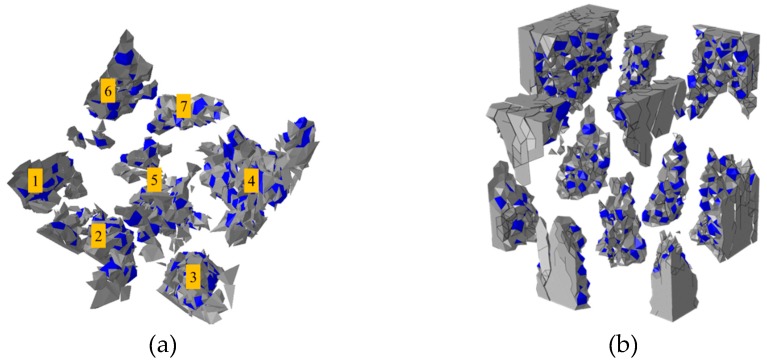
Fragments of the failure concrete specimen: (**a**) the deform shape of the unmoving part which is magnified 20 times, (**b**) the separated fragments of specimen.

By amplifying the deform shape of moving part (unmoving part) 20 times as shown in Figure 12a, it can be found that the concrete specimen has been crushed into several separate fragments (Figure 12b). These fragments are all awl-shaped with one side wide and the other side sharp. Through the shape of the crushed fragments, it can be inferred that at the initial stage of failure, the awl-shaped fragments are shaped by the development of cracks and not totally separated yet. With the proceeding of loading process, these unseparated awl-shaped fragments of moving part wedge the ones of unmoving part, which finally causes the concrete being totally crushed into separate fragments.

#### 3.2.2. High-Friction (HF) Condition

**Figure 13:**
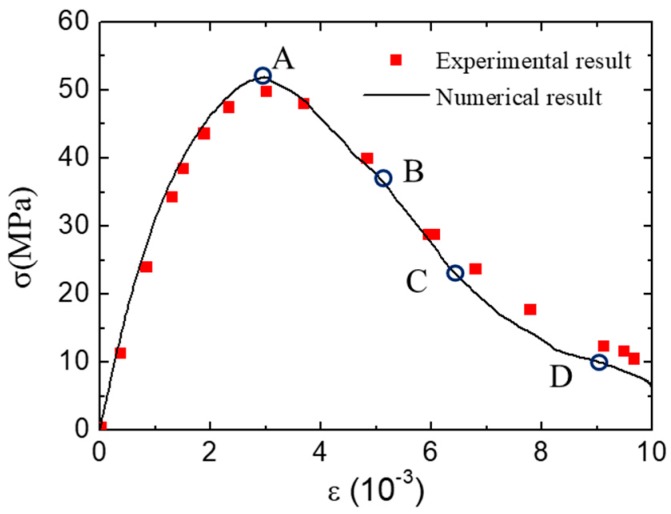
Stress-strain curve of concrete under uniaxial compression in high-friction (HF) condition.

The average stress-strain curve in the HF condition is shown in Figure 13. The numerical result is similar to the experimental one [46] in terms of peak stress and the curve shape. The strength of concrete is much higher than the one in the LF condition. To analyze the fracture patterns of concrete during the loading process, four typical states are chosen and marked in the stress-strain curve (Figure 13).

**Figure 14:**
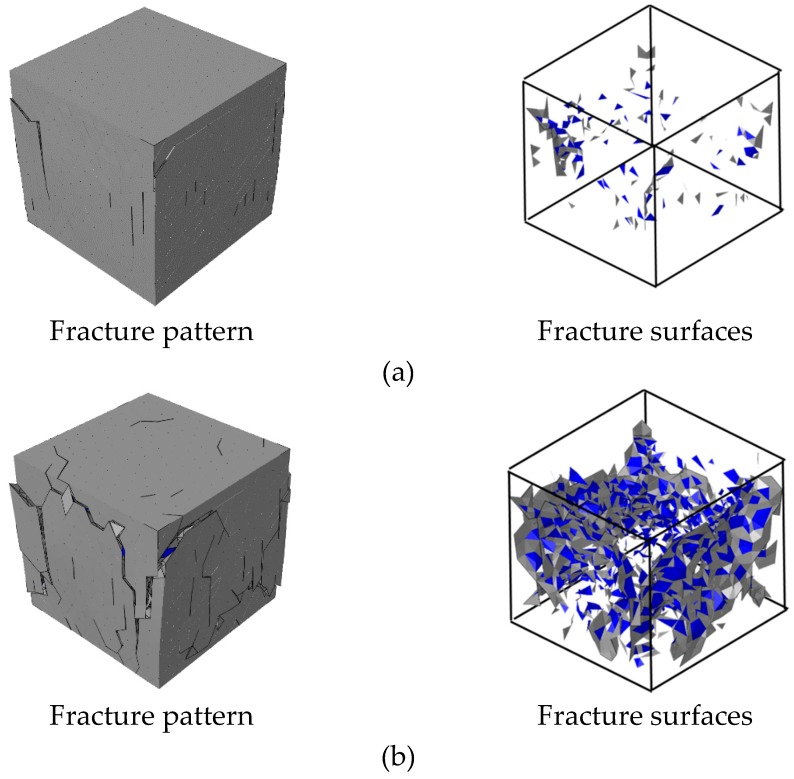

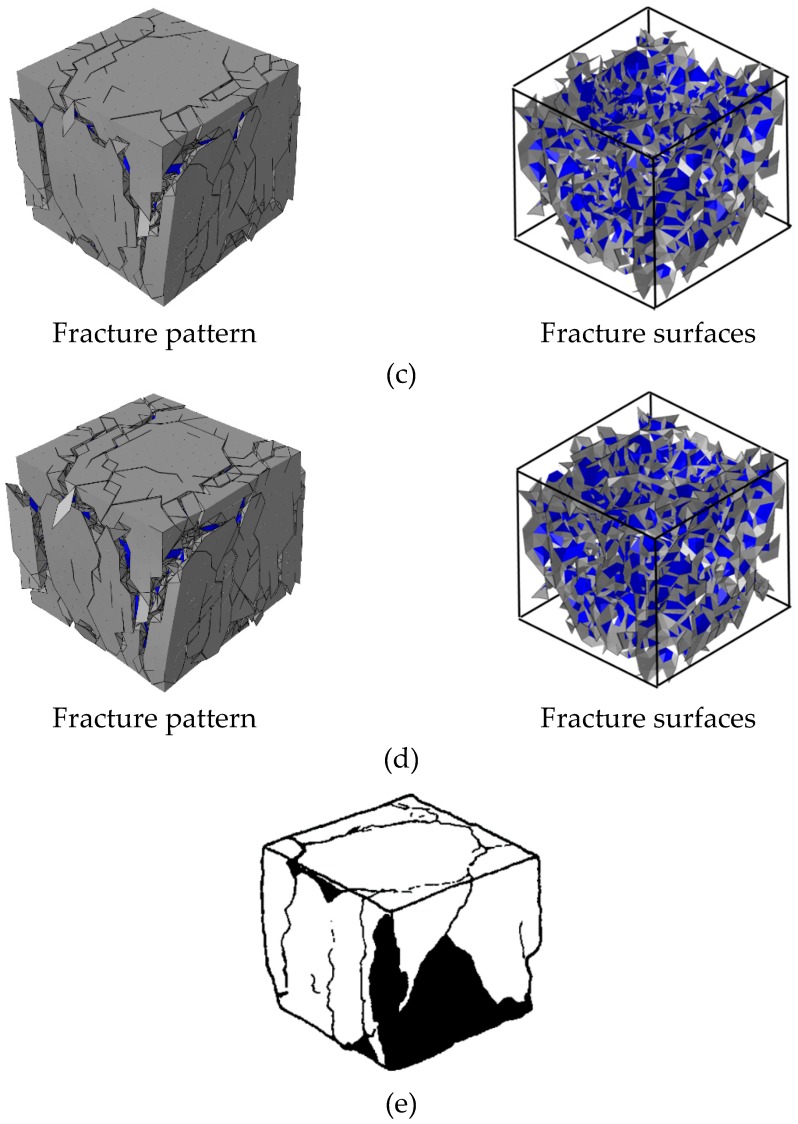
Fracture pattern (aggregate in blue and cement matrix in gray) and fracture surfaces (ITZ in blue and cement matrix interfaces in gray) in different states during the loading process in Figure 13: (**a**) state A, (**b**) state B, (**c**) state C, (**d**) state D and (**e**) the final fracture pattern in experiments [46].

The fracture pattern (deformation is magnified 5 times) and the fracture surfaces of concrete in four different states are shown in Figure 14. At the initial stage of the fracture process, the cracks mainly occur near the four free surfaces, while the surfaces contacted to the rigid plate remain complete. With the increase of loading, the cracks gradually developed into the interior of the concrete specimen, and the concrete fragments near the free surfaces were gradually split from the concrete specimen. The surfaces contacted to the rigid plate always remained relatively complete compared to those in the LF condition. The final fracture pattern shows an agreement with the experimental one.

**Figure 15:**
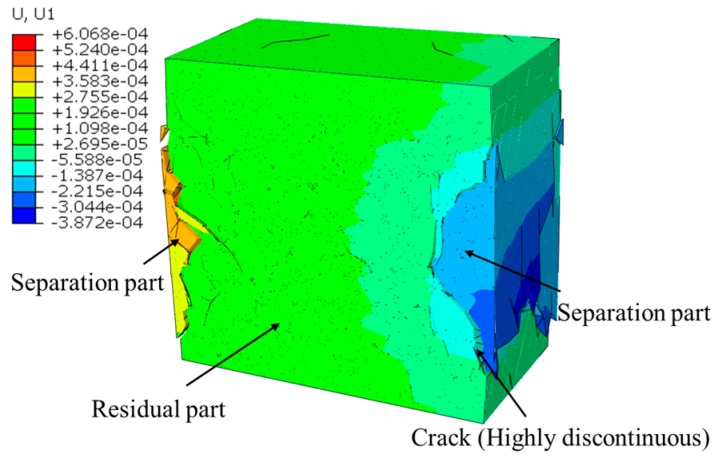
The displacement nephogram whose direction is vertical to the loading one.

**Figure 16:**
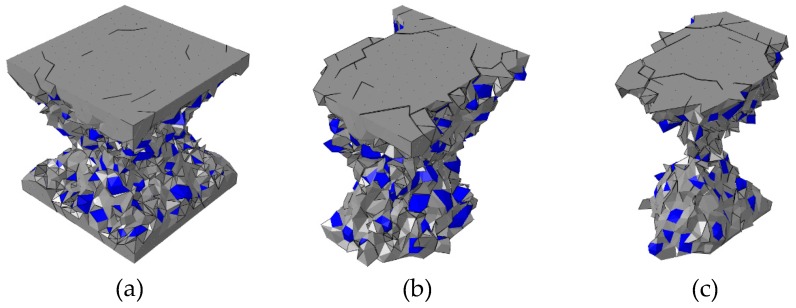
The residual part of the concrete specimen at different loading states in Figure 13: (**a**) state B, (**b**) state C, (**c**) state D.

By observing the nephogram of displacement whose direction is vertical to the loading one (Figure 15), the concrete can be divided into two parts according to the displacement value. One namely residual part has a small displacement, and the other namely separation part has a larger displacement (the displacement value of the separation part is 10 times larger than the residual part). These two parts are separated by cracks with high discontinuity, like the situation in the LF condition. Thus, we can obtain the residual part during the fracture process by deleting the elements with higher displacement values (Figure 16a–c). It can be seen that as the fracture process proceeded, the residual part gradually shrank with the splitting of the separation part. However, due to the influence of surface friction, the residual part still remained dumbbell shape with a thin middle part and two thick ends.

#### 3.2.3. Energy Analysis

**Figure 17:**
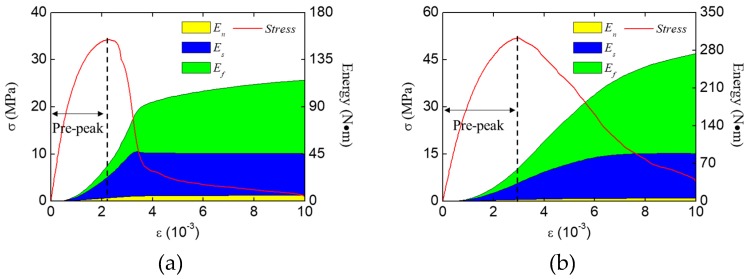
Energy evolution of the concrete specimens under compression in different loading conditions: (**a**) LH condition, (**b**) HF condition.

The fracture modes of concrete under compression are investigated through the energy analysis. The energy evolution of concrete specimens during the loading process are shown in Figure 17. It can be seen that the shear stress work and the friction stress work dominate the energy consumption during the whole loading process, and the normal stress work always keeps a low value. At the final stage of failure, the friction stress work is higher than the shear stress one in both loading condition. As the friction can only occur when the cracks are closed and under compression, it can be inferred that in the compression condition, the compression-shear fracture mode is the main fracture mode inside the concrete, and the tensile-shear fracture mode is not so important.

**Figure 18:**
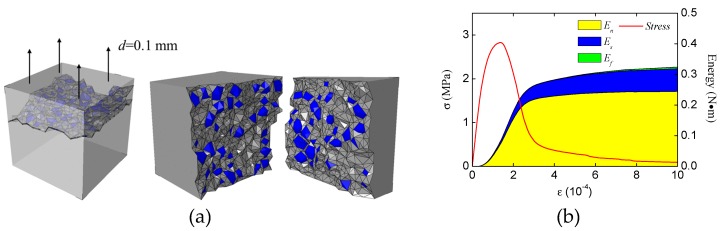
Fracture pattern and energy evolution of the concrete specimen under uniaxial tension: (**a**) fracture pattern and main fracture surfaces, (**b**) energy evolution.

For comparison purposes, we have carried out a group of numerical experiments of concrete specimens under uniaxial tension. The final fracture pattern and the energy evolution of concrete specimens are shown in Figure 18. Unlike the compression condition, the normal stress work dominates the energy consumption during the whole loading process, and the shear stress work only occupies a small proportion of energy consumption in the post-peak stage. Also, the friction work has no contribution during the whole loading process. That is also why the previous work [33,34,35] can obtain reasonable results without considering the friction effect. Thus, according to the energy consumption proportion during the loading process, we can identify the main fracture mode of concrete structures (e.g., concrete beam or concrete column) in the future studies using the proposed numerical method. When the shear and friction stress work account for a large proportion of energy consumption, the main fracture mode is compression fracture. When the normal stress work dominates the energy consumption, the main fracture mode is tension fracture. It is worth noting that there are still many complicated fracture modes of concrete needing analysis, and we will analyze them in future studies.

**Figure 19:**
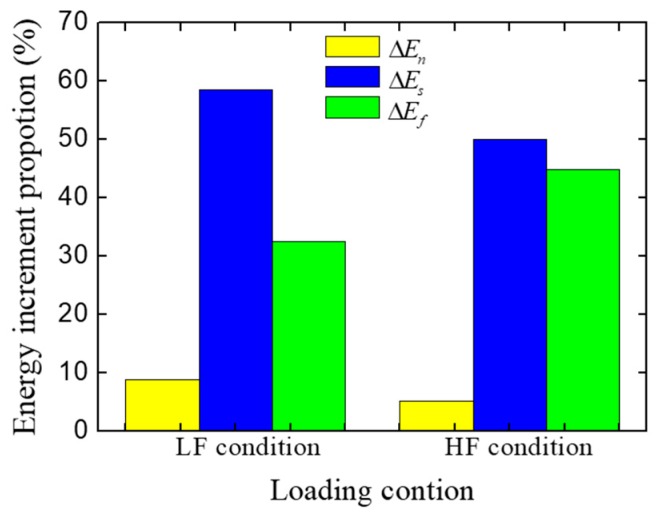
Energy increments proportion of the concrete specimen in different loading conditions at the pre-peak stage.

To investigate the effect of loading condition (LF, HF) on the compressive strength of concrete in terms of energy, the energy increments at the pre-peak stage (Figure 17) are extracted and listed in Table 2. Also, the proportion of different kinds of energy increment are shown in Figure 19. It can be seen that the proportion of the shear stress work increment in the HF condition is significantly lower than that in the LF condition, while the proportion of the friction stress work increment in the HF condition is higher than that in the LF condition. The reason why these differences exist is the restrain effect of the loading surface friction. Due to the restraint of the loading surface, the concrete specimen in the HF condition cannot separate freely as that in the LF condition. For this reason, more cracks inside the concrete are closed and subjected to compression in the HF condition, which causes a higher proportion of the friction stress work and a lower proportion of the shear stress work.

### 3.3. Effect of Specimen Height

**Figure 20:**
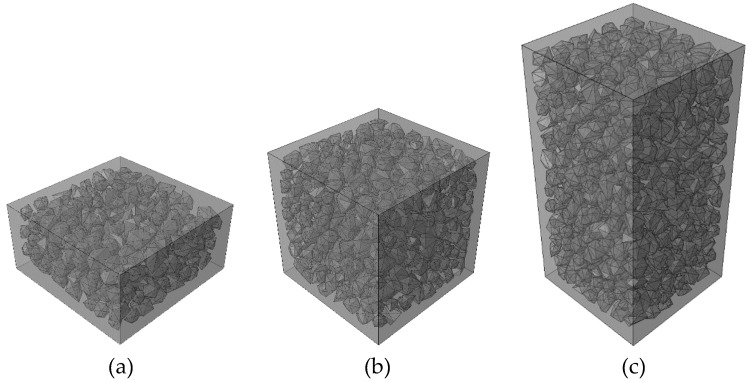
Concrete specimens with the different heights: (**a**) 50 mm, (**b**) 100 mm, (**c**) 200 mm.

To investigate the effect of the specimen height on the fracture pattern and the mechanical behavior of concrete, three kinds of the concrete specimen with different height (50 mm, 100 mm, 200 mm) were chosen for the numerical experiments in the HF condition as shown in Figure 20. All experiments were ended when the displacement *d* = 1 mm.

#### 3.3.1. Fracture Pattern and Mechanical Behavior

**Figure 21:**
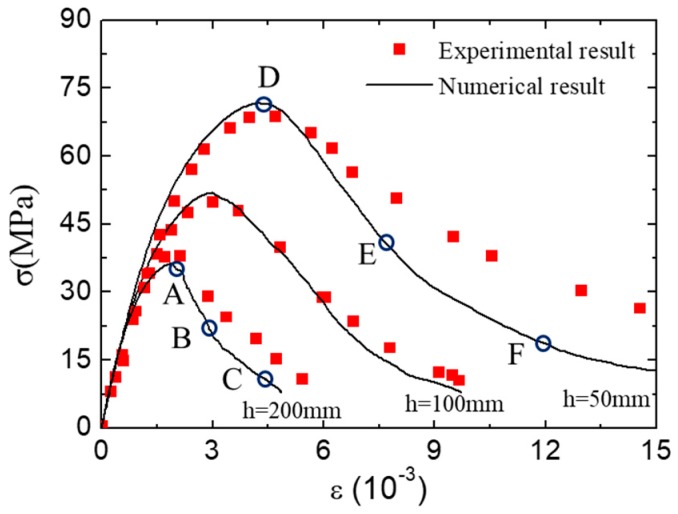
Stress–strain curve of concrete specimens with different heights.

Figure 21 shows the average stress-strain curves of the concrete specimens with different heights. The strength of the concrete specimen decreases with the increase of the specimen height, but the shape of the stress–strain curves is basically unchanged. The numerical stress-strain curves fit well with the experimental one, especially in the pre-peak stage. To investigate the fracture pattern of concrete specimens during the loading process, three typical states were chosen from the concrete specimens with different height (height = 50 mm, 200 mm).

**Figure 22:**
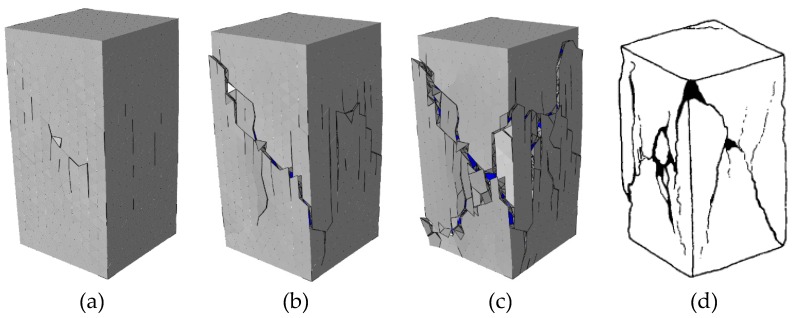
Fracture pattern (aggregate in blue and cement matrix in gray) of the concrete specimen with 200 mm height at different states in Figure 20: (**a**) state A, (**b**) state B, (**c**) state C, and (**d**) the final fracture pattern in experiments [46].

The evolution of concrete fracture pattern (deformation is magnified 5 times) with the specimen height = 200 mm is shown in Figure 22. The final fracture pattern is similar to that obtained from experiments [46]. Due to the restraint effect of the loading surfaces, the cracks mainly occur at the middle part along the height direction, and the loading surfaces always remain complete during the loading process.

**Figure 23:**
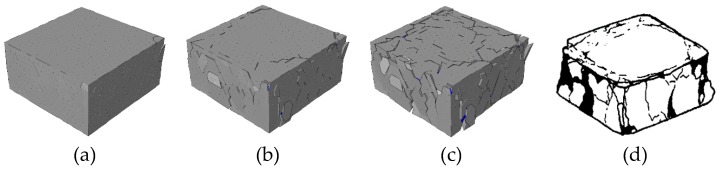
Fracture pattern (aggregate in blue and cement matrix in gray) of the concrete specimen with 50 mm height at different states in Figure 20: (**a**) state D, (**b**) state E, (**c**) state F, and (**d**) the final fracture pattern in experiments [46].

The evolution of concrete fracture pattern (deformation is magnified 5 times) with the specimen height = 50 mm is shown in Figure 23. Due to the small specimen height, the fracture region of the concrete specimen is strongly affected by the restraint of the loading surfaces. Thus, the deformation of this specimen is smaller than the other two kinds with different height, and the cracks mainly occurs near the free surfaces.

#### 3.3.2. Energy Analysis

**Figure 24:**
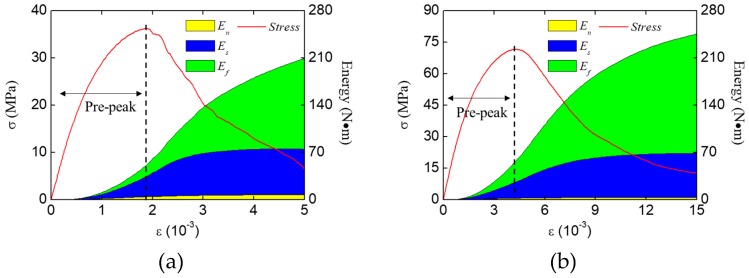
Energy evolution of the concrete specimen under compression with different specimen heights: (**a**) 200 mm, (**b**) 50 mm.

The fracture modes of concrete with different height are investigated through the energy analysis. The energy evolution of the concrete specimens with different heights (200 mm, 50 mm) are shown in Figure 24. The energy evolution regulation of these two specimens are similar to that with the height = 100 mm (Figure 17b). The normal stress work still has little effect on the energy consumption, while the shear stress work and the friction stress work always play a dominated role during the fracture process. However, the proportion of friction stress work increases with the decrease of the specimen height.

**Figure 25:**
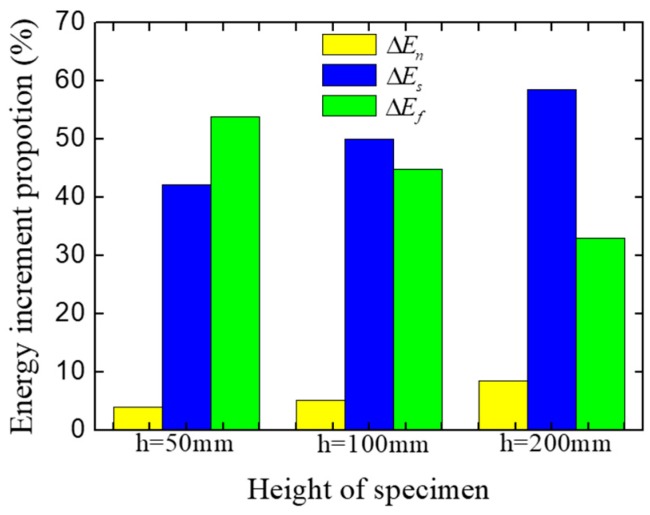
Energy increments proportion of the concrete specimen with different specimen heights at the pre-peak stage.

The energies increment at the pre-peak stage (Figure 24) were extracted and are listed in Table 3, and the proportion of different kinds of energy increments are shown in Figure 25. As shown in results, the proportion of friction stress work decreases with the increase of the specimen height, while the proportion of the normal stress work and the shear stress work increase. This change means the restraint effect of loading surfaces has a limited effect range. when the fracture region is inside the effect range of the restraint of the loading surfaces, the free separation of cracks would be restrained, which causes a higher proportion of friction stress work; but when the fracture region is far enough to get rid of the restraint of loading surfaces, the cracks can separate freely, and the fracture process at the pre-peak stage would be similar to that in the LF condition at the pre-peak stage. For example, the strength and the energy increments proportion of the specimens with 200 mm height in the HF condition (Figure 24a and Figure 25) are basically same as the one in the LF condition (Figure 17a and Figure 19). However, with the development of the cracks, by which the fracture region would be affected by the restraint effect of loading surfaces, the fracture pattern and the mechanical behavior would be different from the one in the LF condition at the post-peak stage.

## 4. Conclusions

A 3D numerical model of concrete mesostructure is proposed based on Voronoi tessellation and cohesive elements. A modified constitutive is established to characterize the complex behavior of the potential fracture surfaces. Based on the proposed model, the effect of the loading condition and the height of the concrete specimens under uniaxial compression were investigated by analyzing the fracture patterns and the energy increments proportion. Several conclusions can be obtained as follows:

(1) The mechanical behavior and the fracture patterns of concrete under compression obtained from the numerical results shows an agreement with that obtained from experiments, which means the proposed model in this paper is suitable for the mesoscopic study of concrete.

(2) The energy consumption proportion can be used to identify the fracture mode of concrete during the fracture process. The compressive fracture of concrete is always accompanied by high shear and friction stress work, and the tensile fracture is accompanied by high normal stress work.

(3) The effect of loading surfaces restraint on the compressive strength can be estimated by the proportion of the energies increment at the pre-peak stage: a higher proportion of the friction stress work and a lower proportion of the shear stress always cause a higher compressive strength.

In this study, we have established a 3D numerical model and used it to analyze the fracture behavior of concrete under compression. However, there are still many things worth studying based on this research. First, the aggregate generated in this paper is based on pure theory, and we can try to replace it with more realistic aggregates reconstructed by CT scanning in further studies. Secondly, there are still many factors that can affect the mechanical behavior of concrete such as the loading form, the loading rate, the aggregate/cement content, and the strength range of concrete. These factors are meaningful to the design of concrete and worth investigating in further studies.

## Figures and Tables

**Table 1:** The size distribution of aggregates in experiment [46].

Grain Size (mm)	Unit Content (kg/m^3^)	Volume Content (%)
8~4	540	19.29
4~2	363	12.91
2~1	272	9.71
1~0.5	272	9.71
0.5~0.25	234	8.35

**Table 2:** Energy increments and proportion of the concrete specimen in different loading condition at the pre-peak stage.

Loading Condition	Energy Type	Energy Value (N∙m)	Energy Proportion (%)
LF condition	$\Delta E_{n}$	3.31	8.88
	$\Delta E_{s}$	21.85	58.57
	$\Delta E_{f}$	12.14	32.55
HF condition	$\Delta E_{n}$	3.12	5.12
	$\Delta E_{s}$	30.47	49.98
	$\Delta E_{f}$	27.37	44.90

**Table 3:** Energy increments and proportion of the concrete specimens with different specimen heights at the pre-peak stage.

Height of Specimen	Energy Type	Energy Value (N∙m)	Energy Proportion (%)
200 mm	$\Delta E_{n}$	4.31	8.49
	$\Delta E_{s}$	29.72	58.51
	$\Delta E_{f}$	16.77	33.00
50 mm	$\Delta E_{n}$	2.26	3.96
	$\Delta E_{s}$	24.05	42.14
	$\Delta E_{f}$	30.77	53.90
